# Aesthetic Chin Retrusion Enhancement With Non-animal Stabilized Hyaluronic Acid-High Density (NASHA-HD™) Filler

**DOI:** 10.7759/cureus.111156

**Published:** 2026-06-19

**Authors:** Marcus H A Morais, Cássio Guimarães, Francine Papaiordanou, Stephanie Oliveira, Alessandra Nogueira, Rafael Tomaz Gomes

**Affiliations:** 1 Medicine, Universidade Federal De Minas Gerais, Belo Horizonte, BRA; 2 Dermatology, Clinical Practice, Belo Horizonte, BRA; 3 Dermatology, Clinical Practice, Sao Paulo, BRA; 4 Medical Affairs, Galderma, Sao Paulo, BRA

**Keywords:** chin, g prime, hyaluronic acid dermal fillers, lower face, nasha-hd™

## Abstract

Background: Chin projection is a critical component of lower face aesthetics, influencing facial balance, youthfulness, and sexual dimorphism.

Methods: This retrospective case series provides short-term, real-world observations on the use of Non-animal Stabilized Hyaluronic Acid - High Density (NASHA-HD™) injectable for chin augmentation as part of a combined lower-face hyaluronic acid (HA) filler treatment approach. Each patient received 1 mL of High-Animal Stabilized Hyaluronic Acid (HASHA™) administered as supraperiosteal boluses in the chin, distributed according to the patient's needs. For a comprehensive facial approach, complementary injections of HA fillers based on Optimal Balance Technology (OBT^™^)and NASHA®technologies were performed in the mid and lower face to harmonize facial proportions and enhance overall contour.

Results: Results demonstrated high patient satisfaction, natural aesthetic outcomes, a natural look on the chin, and a favorable safety profile with only mild and transient adverse events. Independent Global Aesthetic Improvement Scale (iGAIS)-physician assessment demonstrated global aesthetic improvement and enhanced facial proportionality in most patients. This holistic approach achieved satisfactory projection and contour enhancement using low product volumes.

Conclusions: This article underscores the importance of using high G′ fillers for structural support, and flexible fillers for dynamic zones, particularly in the diverse population, which presents a wide range of facial morphologies requiring personalized aesthetic approaches.

## Introduction

In the lower face, the chin position is recognized as an essential element for overall facial balance and lower face contour definition, contributing to the perception of youthfulness and sexual appeal [1,2]. Age-related factors, such as bony loss, may lead to jowling and reduced chin projection. In addition, a genetically underdeveloped or recessed chin is also associated with a less esthetically pleasing facial profile and can contribute to early loss of definition of the jawline [3,4]. Treating lower face discrepancies aims to restore a youthful shape and create smooth transitions from the midface to the mandible and neck. The goals of chin augmentation procedures include achieving the appropriate facial height, complementing nasal projection, and defining the mandibular line [1]. Optimized chin projection may also improve the appearance of adjacent areas, such as pre- and post-jowl and signs of aging in the neck, including the submandibular area [2].

Amongst dermal fillers, hyaluronic acid (HA)-based gels are increasingly popular due to their immediate and visual effects on the chin, their demonstrated safety, and ease of use. Surgical augmentation of the chin has declined in popularity in the last few decades; consequently, the use of HA dermal fillers has been rising as an effective non-surgical alternative, which offers a safer and temporary option for correcting mild-to-moderate chin retrusion [5]. As the primary function of a tissue filler is to fill skin wrinkles and folds and restore facial volumes with adequate biointegration, as well as promote facial projection and replenishment, their mechanical behavior is a key feature of their clinical use and performance. Notably, HA fillers enable the three-dimensional shaping of the chin, with individual adaptation for each patient [6].

In this context, Non-animal Stabilized Hyaluronic Acid - High Density (HA_SHA_ is an HA dermal injectable produced with a new NASHA-HD™ technology, which is an evolution of the NASHA® platform that incorporates more HA molecules and employs a more efficient cross-linking while maintaining the same low degree of modification. This new technology results in a product being even firmer compared with all other NASHA® gels. HA_SHA_ has an HA-concentration of 25 mg/mL and G' of 916 Pa (0.1 Hz) and was specifically developed for lower face shaping and temporary chin augmentation, optimizing volumes to be injected deeply on the bone supraperiosteal plane [7]. These rheological properties may lead to reducing biodegradation and increasing durability [8-10]. In 2023, HA_SHA_ was approved in Canada for temporary augmentation in the chin region and in 2024 in Brazil.

Scientific data in diverse populations providing real-world insights are scarce in the literature. As mixed-heritage people, Brazilian individuals exhibit considerable variation in lower facial morphology, particularly in the chin and mandible, influenced by sex, age, and ethnicity, including distinct sagittal and vertical skeletal patterns [11]. White Caucasian Brazilians tend to have a higher mandibular symphysis with lesser anterior projection, while African-American Brazilians typically present a more protruded maxilla and mandible, smaller chin prominence, and greater bimaxillary dentoalveolar protrusion [12]. When compared to Asian Brazilian male individuals, those men present a more acute nasolabial angle and greater maxillary and lip protrusion [13]. These variations highlight the need for individualized treatment planning to achieve a balanced facial aesthetics and optimize outcomes with HA fillers such as HA_SHA_.

Hence, this retrospective case series provides short-term, real-world observations on the use of NASHA-HD™ filler for chin augmentation as part of a combined lower-face HA filler treatment approach in Brazilian patients based on the authors' clinical practice.

## Materials and methods

All patients were assessed and treated by experienced medical injectors in a private office in São Paulo, Brazil, and provided written informed consent prior to participation. All data were collected retrospectively. The ethics approval (CAAE: 90282625.8.0000.0444) was obtained in accordance with Good Clinical Practices and the Declaration of Helsinki [14].

Study sample

Fourteen patients, aged ≥18 years, with aesthetic concerns involving chin projection, who sought aesthetic treatment, were included in this analysis. In addition, midface and lower face features were subjected to a comprehensive facial assessment. For the chin retrusion, patients graded as 1-3 points as the Galderma Chin Retrusion Scale (GCRS scale) [8] were included. Exclusion criteria including patients who have previous permanent/semi-permanent fillers in the area or received any other injectable treatments in the chin region three months prior the procedure, active skin infection, inflammation, pregnant or breastfeeding, autoimmune diseases, bleeding disorders, or anticoagulant use in the last two weeks were not eligible for the treatment.

This evaluation was performed prior to the injection procedure to grade the chin retrusion and guide the clinical diagnosis and the development of an individualized treatment plan.

**Figure 1:**
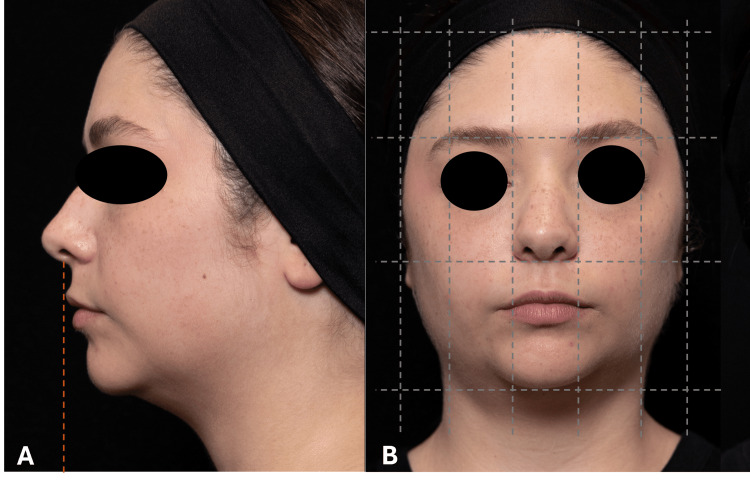
Rationale for proportionality assessment (A) Orange line: nasolabial line. (B) Horizontal thirds and vertical fifths. Equidistant distances from the facial frame to the center.

Post treatment, especially for the chin appearance, the assessment references were the nasolabial line (Figure 1A). The facial proportions and chin scale guided the determination of optimal injection points and the filler volume per point on the mid and lower face, including the chin [15].

In addition, facial balance was assessed using horizontal thirds and vertical fifths. Briefly, the face was divided into five equal segments - horizontal thirds lines in trichion-glabella, glabella-subnasale, subnasale-menton. Vertical fifths were drawn in the facial frame, lateral and medial canthus, ala of nose (Figure 1B), while facial thirds and fifths represent a well-established framework for the systematic evaluation of facial proportions, symmetry, and aesthetic balance. They are extensively utilized in clinical aesthetic planning and are regarded as a gold standard in evidence-based facial anthropometry.

Outcomes

The main outcome was the five-point Global Aesthetic Improvement Scale (GAIS) graded at 28-35 days post treatment (days 28-35), in comparison with D0 by the comparison of the 2D standardized pictures. Based on the patient photos, the independent evaluator (iGAIS) focused on the lower face, considering the categories of improvement were as follows: -1: "Worsened", 0: "No change", 1: "Improved", 2: "Very improved", and 3: Very much improved".

As a secondary outcome, the facial proportions, such as nasolabial line (Figure 1A), horizontal thirds, and vertical fifths (Figure 1B), were considered with outcomes. The facial proportions were categorized as "no change", "improved", or "very much improved" [16].

Subject satisfaction was also assessed, and participants were asked to rate their improvement of the aesthetic profile, natural appearance, and overall satisfaction with the treatment at days 28-35.

Safety 

Safety data were collected by follow-up calls within 14 days after the procedure, including the onset, duration, and outcome of these events.

Treatment

The treatment procedure was conducted in a single and individualized treatment session according to patient needs, considering midface and lower-face contour improvement and chin augmentation. Final decisions regarding technique and product allocation were made at the physician's discretion.

**Figure 2:**
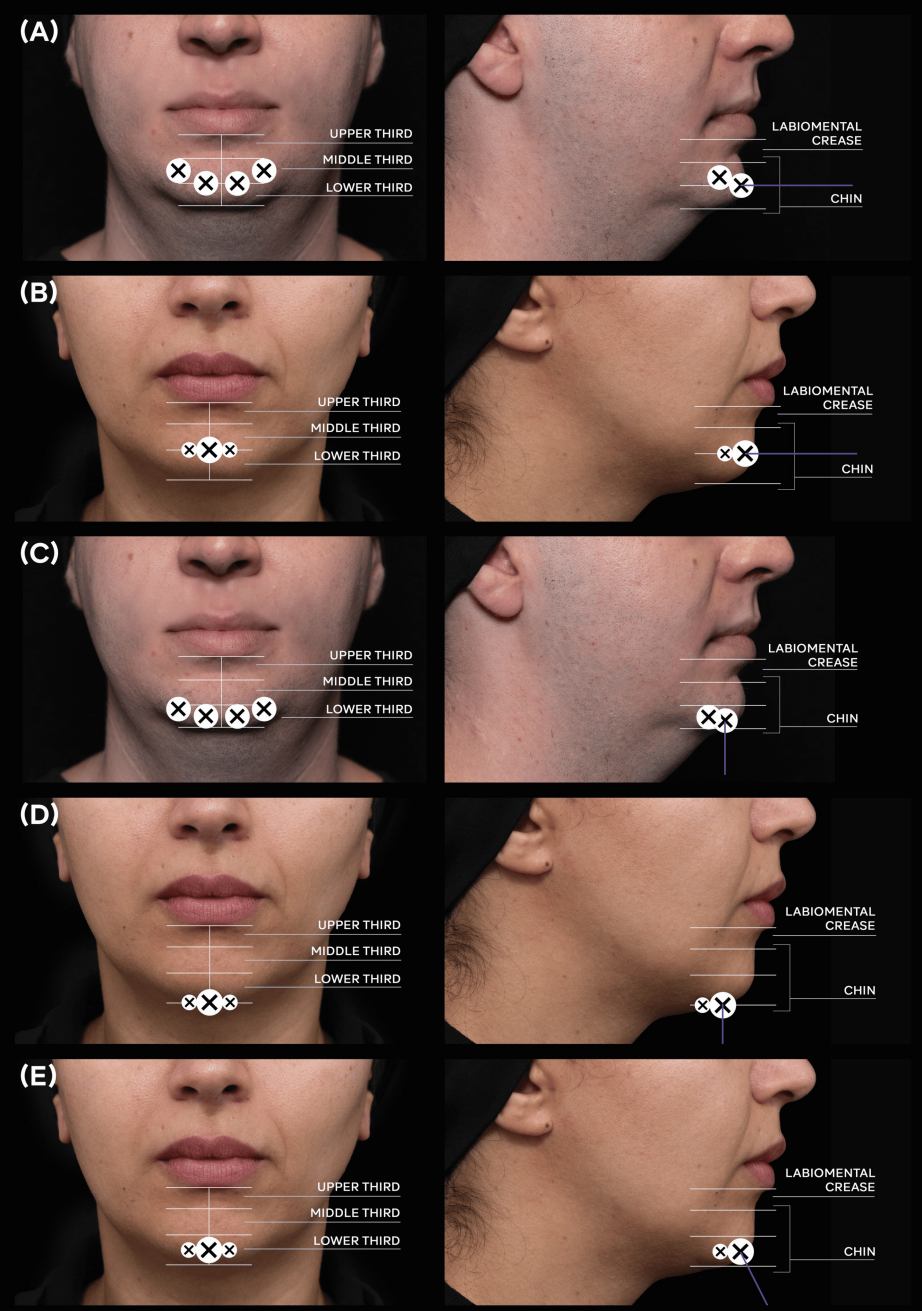
Clinical landmarks for chin assessment and treatment Illustrating key injection points: white line, assessment markings; dark cross, entry points; white circle, bolus injections; blue line, direction of the injection. Panels: (A) male chin projection, (B) female chin projection, (C) male chin elongation, (D) female chin elongation, and (E) female chin projection and elongation. Vector graphics were created by Juliana Marques (graphic designer) using Adobe Illustrator. Written consent was obtained from the patients for publication in an open-access journal.

HA fillers with distinct rheological properties and technologies, including OBT™ (HA_DEF_), NASHA® (HA_LYF_), and NASHA-HD™ (HA_SHA_), were selected based on the facial anatomical regions, tissue biomechanics, and skin thickness of patients. HA fillers with greater flexibility and tissue integration (OBT™) were selected for areas of higher mobility, while firmer formulations (NASHA® and NASHA-HD™) were used in regions requiring additional projection and definition [17,18]. HA_SHA_ was injected exclusively in the chin using needle‑based bolus injections placed at the supraperiosteal plane, with a previous 10 seconds of aspiration, following the technique described by Nikolis et al. [9]. The number of injection points ranged from one to four, depending on the desired correction. For chin projection, a bolus was placed on the midline, and/or one to two boluses were placed bilaterally adjacent to the midline. For elongation, the bolus was positioned centrally along the lower third of the mandibular border or distributed bilaterally along this border (Figure 2).

The jawline treatment approach combined both needle and cannula techniques to optimize precision and product distribution. The pre‑jowl and mentolabial fold were treated with a cannula using linear retrograde fanning within the subcutaneous layer. In selected patients, bolus injections with a needle were administered in the lower portion of the marionette lines. For the malar region and the pyriform aperture, HA filler was injected subcutaneously either with a cannula or at the supraperiosteal plane with a needle. Figure 2 illustrates the overall distribution of HA fillers across the potential facial regions treated.

Patients had not received any other injectable treatments in the chin region during the three months preceding the procedure. Post-treatment procedures included guiding patients on standard post-treatment care, including medication prescription to reduce possible discomfort when needed.

Statistical analysis

Data were analyzed using descriptive statistics. Qualitative variables were presented as absolute and relative numbers, and quantitative variables were presented as mean and standard deviation. Microsoft Office Excel® (Microsoft® Corp., Redmond, WA) was used to calculate descriptive statistics and generate charts.

## Results

Data from a total of 14 patients were collected. They were healthy men and women treated with HA fillers and followed for up to 35 days. Most of them were female (85.7%), with a mean age of 39.1 years and an age range of 20-58 years. The most common Fitzpatrick skin types were III (28%) and IV (35%), and most patients presented with GCRS grade 1 or grade 2 chin retrusion at baseline assessment (Table 1).

**Table 1:** Social-demographic and clinical characteristics at baseline (n = 14) GCRS, Galderma Chin Retrusion Scale; SD, standard deviation

Demographic/Characteristic	n (%)
Age (years), mean (SD)	39.1 (11.7)
Female	12 (85.7)
Ethnicity	
Latin American	5 (35.7)
Caucasian	6 (42.8)
African American	3 (21.4)
Fitzpatrick Skin Phototypes	
I	1 (7.1)
II	3 (21.4)
III	4 (28.6)
IV	5 (35.7)
V	1 (7.1)
GCRS Score	
1	6 (42.8)
2	6 (42.8)
3	2 (14.3)

All patients received 1 mL of HA_SHA_ in the chin as supraperiosteal boluses distributed across two to four injection points. OBT™ and NASHA® HA injectables were used as needed in the mid-face and lower face to enhance structural support and jawline contour, respectively. In this sense, all 14 patients received 1-2 mL of HA_DEF_ through retrograde subcutaneous injections in the pre-jowl, marionette lines, mentolabial fold, or malar region to improve contour and achieve smoother transitions. Similarly, optional injections of HA_LYF_, with up to 1 mL, were administered in 11 patients as supraperiosteal boluses at the pre-jowl and chin to reinforce structural support, as well as in the pyriform region when indicated. Table 2 summarizes the technical considerations for injection procedures in the chin projection and transition areas using hyaluronic acid fillers.

**Table 2:** Technical considerations of injection procedures for chin projection and transition areas* with hyaluronic acid fillers *Transition areas: pre-jowl and marionette lines. **One or two injection points per side of the chin midline or one central injection point with one at each side of the chin midline. *** 90° injection angle in relation to the skin.

Anatomical region	Product	Injection points per side	Injection points in total**	Volume (mL) per injection point/side	Device	Plan	Deposition
Chin	HA _SHA_	1-2	2-4	0.2-0.6	27 G needle ***	Supraperiosteal	Bolus
Pre-jowl	HA _LYF_	-	-	0.2-0.6	25G cannula	Superficial subcutaneous	Retroinjection
	HA _DEF_	-	-	0.2-0.7			Retroinjection
Marionette lines	HA _DEF_	-	-	0.5-0.5	25G cannula	Superficial subcutaneous	Retroinjection
Mentolabial fold	HA _DEF_	-	-	0.1-0.3	25G cannula	Superficial subcutaneous	Retroinjection

**Figure 3:**
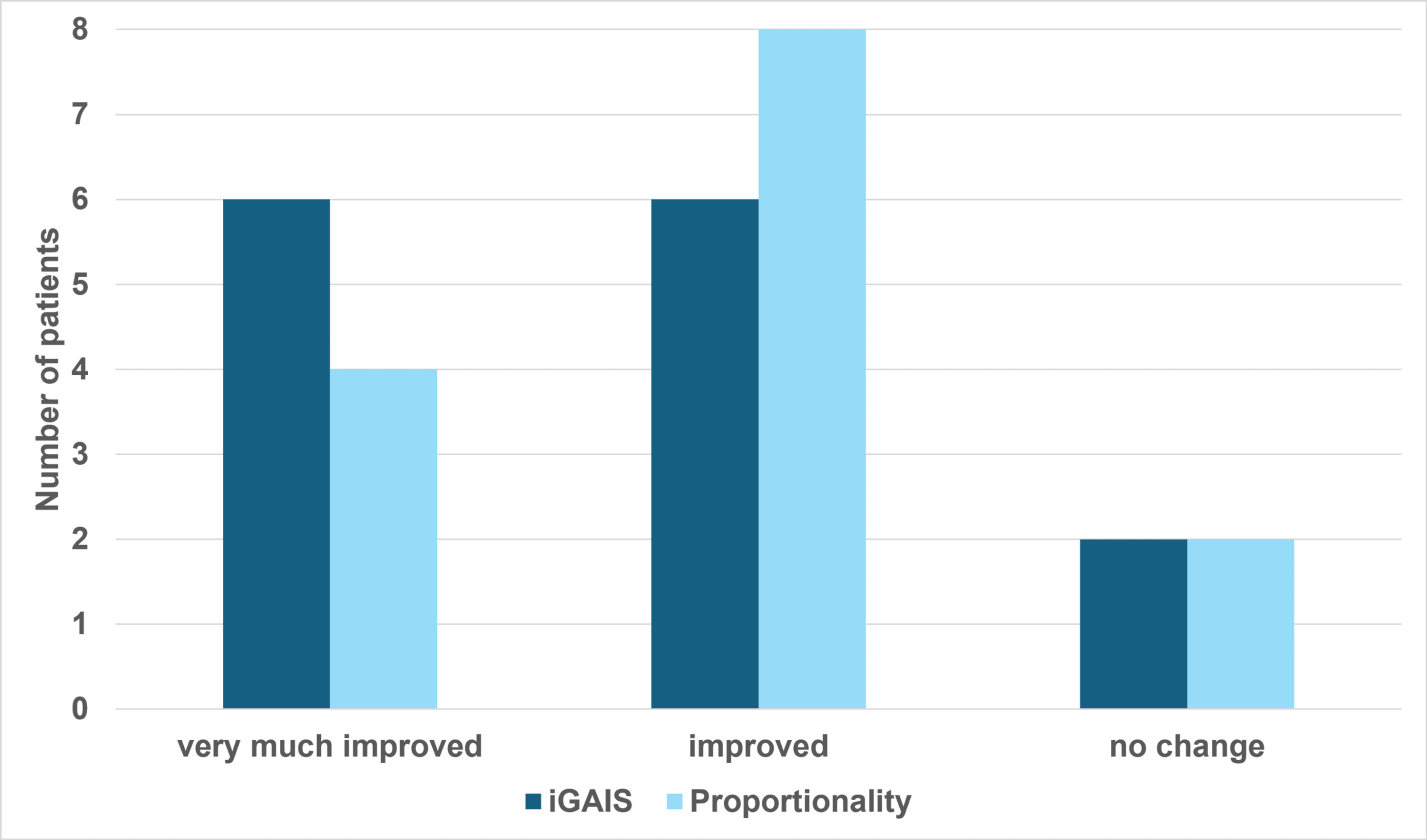
Independent evaluator GAIS (iGAIS) and facial proportionality outcomes at follow-up (28-35 days) based on 2D image evaluations (n = 14) Most patients were classified as "improved" or "very much improved" in both assessments.

**Figure 4:**
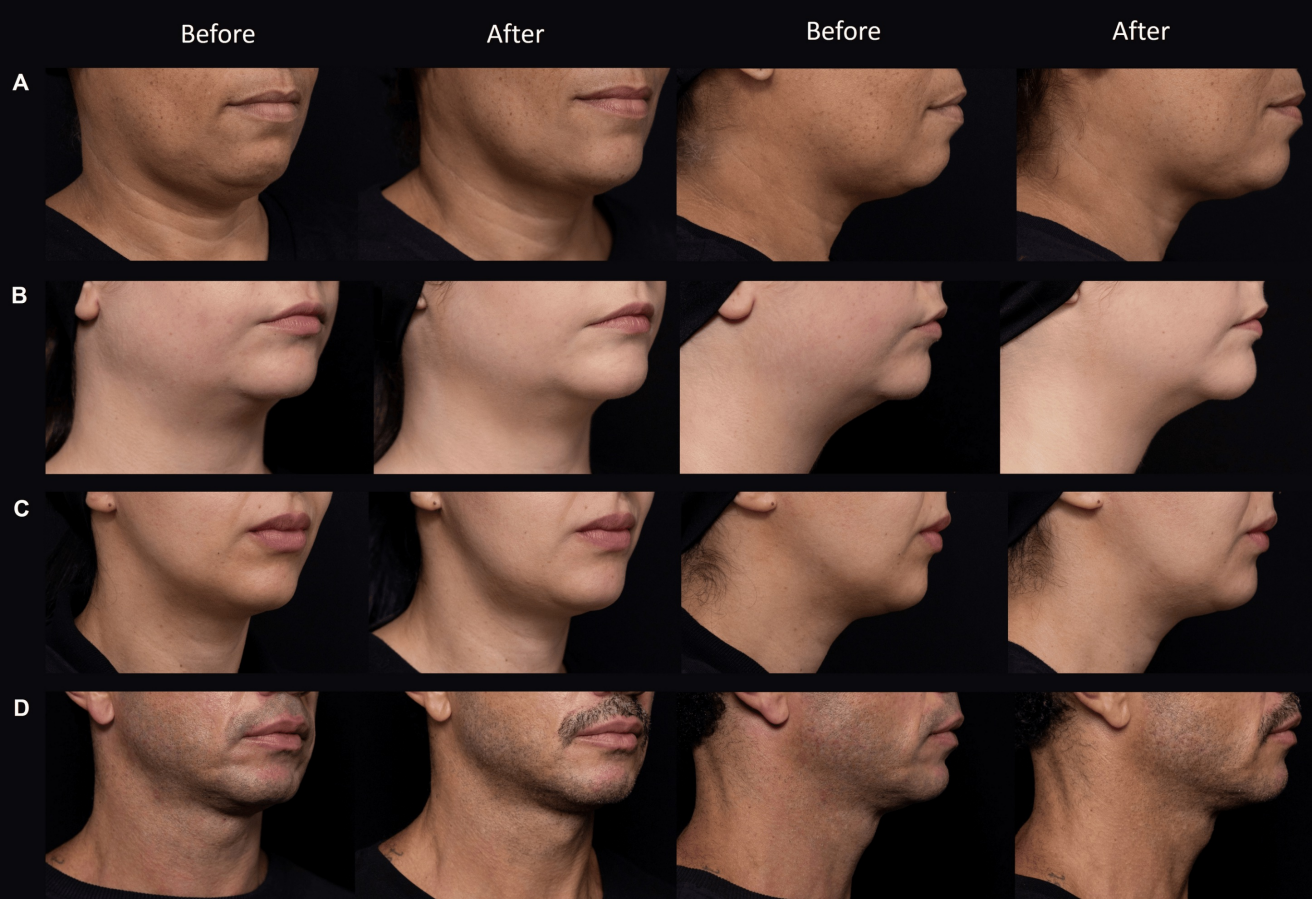
Representative baseline and day 35 post-treatment images of patients with varying genders, ethnic backgrounds, ages, and facial morphologies who underwent lower face rejuvenation Each row displays frontal, 45-degree, and lateral views of the same patient. Treatments included HA_SHA_ for the chin and pre-jowl, HA_LYF_ for the chin or piriform fossa, and HA_DEF_ for the malar area, marionette lines, or pre-jowl, depending on individual needs. A total of 1 mL per product was used in each treated area. (A) 43-year-old female (patient 14); (B) 28-year-old female (patient 08); (C) 29-year-old female (patient 09); and (D) 47-year-old male (patient 12). Written consent were obtained from all patients for publication in an open-access journal.

Clinical improvement was assessed using photographic records taken at the follow-up visit. The independent, blinded evaluator GAIS scores indicated that most (85.7%) patients were classified as "improved" or "very much improved," while only two patients showed no noticeable changes. No patients were rated as "worse" or "very much worse" on the GAIS. Similarly, improvements in facial proportions followed the same trend, with 85.7% of patients rated as "improved" and "very much improved." Only two patients exhibited no changes in facial proportions (Figure 3). Representative pre- and post-treatment images are presented in Figure 4.

The overall subject satisfaction with their appearance was high, and all participants either agreed or strongly agreed that their chin had improved aesthetically and looked natural.

Most patients reported experiencing at least one event within 14 days of the procedure. The events occurred on the same day or the day following the injection, with an average duration of three days, and were mostly related to pain and bruising at the injection site (Table 3). Additionally, 96% patients were classified by patients as mild or moderate in intensity, while one patient (4%) reported intense pain at the injection site, which was attenuated using oral analgesics. No cases of infection, paresthesia, asymmetry, necrosis, or skin discoloration were reported. No serious event was reported. All subjects who experienced any event had fully recovered spontaneously or after taking pain relief medication for a short time within 14 days.

**Table 3:** Subject reported, post-injection events (n = 14)

Post-injection event	n	% patients
Pain at the injection site	8	57.1%
Bruise/hematoma at the injection site	4	28.6%
Edema at the injection site	6	42.8%
Pruritus at the injection site	4	28.6%
Papule/Nodule at the injection site	2	14.3%
Pain during the injection	1	7.1%
Erythema at the injection site	1	7.1%

## Discussion

This paper presents real-world insights on lower facial treatment of Brazilian patients using HA fillers, focusing on chin enhancement with HA_SHA_ injectable. In a Phase 3 trial, HA_SHA_ demonstrated sustained improvement in chin retrusion over 12 months, with high patient and investigator satisfaction and no serious adverse events [8]. As the pivotal study primarily included Caucasian female patients, the present report investigates the first group of Brazilian patients treated with this technology, underscoring population diversity and individualized aesthetic needs. Overall, the treatment demonstrated highly satisfactory clinical outcomes, with a favorable safety profile and efficient volumizing effect, achieving optimal results with low filler volume. Evaluating the performance of aesthetic products in a highly mixed and diverse population with skin of color, such as the Brazilian, is essential to ensure effective and safe treatments [19]. This genetic diversity is reflected in a wide range of facial morphologies and phenotypes, particularly in the lower third of the face, where significant differences are observed in chin projection, mandibular contour, and soft tissue distribution. These anatomical variations necessitate personalized approaches when using dermal fillers, considering ethnic and individual differences to achieve natural and harmonious results.

The treatment planning was determined by the physician's discretion based on individual patient needs. All patients received chin injections using supraperiosteal boluses at two to four points, facilitated by the 19 mm needle provided with the NASHA-HD™ injectable. High G′ fillers, such as the one used in this study, are preferred for chin augmentation due to their ability to withstand compression from overlying tissues when injected deeply at the periosteum [9,20]. In line with expert recommendations, initial treatment volumes should remain conservative, typically ranging from 1 to 2 mL, to minimize the risk of overcorrection, patient discomfort, and adverse events [9]. In fact, the authors noted that HA_SHA_ provided excellent tissue projection even with low injection volumes, which was consistent with the high levels of patient satisfaction observed post-procedure. These findings support the product's favorable safety profile and its ability to achieve natural-looking results with minimal filler use.

The chin has a rich vascular network, requiring caution to avoid intravascular injection or compression of vessels, nerves, or other structures [9]. Therefore, products that respond rapidly to hyaluronidase, such as NASHA-HD™ injectable, are particularly recommended. An in vitro study has shown that both HA_SHA_ and HA_LYF_ were rapidly degraded by hyaluronidase of different origins and concentrations, unlike other HA technologies such as Vycross [21]. Although HA_SHA_ has a higher HA and G’, its efficient crosslinking and low degree of modification may contribute to its favorable responsiveness to hyaluronidase and safety profile, which aligns with the authors' clinical perceptions.

Chin projection and length can be enhanced by targeting the pogonion and menton areas, respectively. Different approaches are recommended based on treatment aim (projection, elongation, or both), and if the purpose is to achieve a traditional feminine or masculine appearance [2,9]. Male patients could be injected at four points to achieve both satisfactory elongation and projection. On the other hand, the protocols in female patients varied depending on their needs but were mostly performed as midline bolus injections, followed by two collateral points if more projection was necessary. Detailed information on chin injection protocols using HA_SHA_ is presented in Nikolis et al. [9].

Besides the correction of the chin area, optimal lower face rejuvenation requires a three-dimensional approach, also addressing adjacent structures, such as the oral commissures, mental crease, marionette lines, pre- and post-jowl sulci, and the mandibular line, angle, and ramus [2,22]. For that reason, the researchers were also provided with two other fillers from different complementary technologies, NASHA® and OBT™. While NASHA® fillers are firmer, with high G’, developed for projection and focal tissue integration, OBT™ fillers are softer and flexible, developed for contour and natural expression in dynamic areas of the face [17,23]. The use of a range of HA fillers allows injectors to achieve a holistic, natural-looking result. In this series, the more flexible fillers with dynamic properties (HA_DEF_, OBT™ technology) were predominantly injected subcutaneously using either a needle or cannula in areas such as the labiomental crease and marionette lines to ensure smooth transitions between treated regions. To complement this approach and enhance lower face definition, the firmer filler with lifting properties (HA_LYF_, NASHA® with high G′) was administered either as boluses or subcutaneously with a needle in areas including the pre-jowl, mandibular angle, and body, providing structure and contour. The combination of these three HA fillers can be considered a comprehensive aesthetic treatment strategy for the lower face.

The small sample size, short follow-up period, and the lack of a comparison group may represent potential biases in this study. Nevertheless, this article provides valuable real-world insights into the safety and effectiveness of HA_SHA_, used in combination with complementary filler technologies for a comprehensive and integrated facial aesthetic approach. Future studies with larger cohorts are warranted to confirm these findings.

## Conclusions

The use of HA_SHA_ for chin enhancement in Brazilian patients yielded favorable clinical benefits, a good safety profile, and high patient satisfaction, with conservative product volumes, which is especially relevant given the facial diversity of the Brazilian population. However, these findings should be interpreted with caution due to the small sample size, short follow-up, and lack of a comparison group.

The high G′ properties of NASHA® and NASHA-HD™ fillers make them suitable for deep injections, offering projection and structural definition. In combination with an OBT™ filler, used in more dynamic areas to ensure smooth transitions, this approach enabled a comprehensive and harmonized treatment of the lower face tailored to each patient's anatomical needs. Larger, longer-term studies with a control group are needed to confirm these results.
